# Financial progress, inward remittances, and economic growth in Bangladesh: Is the nexus asymmetric?

**DOI:** 10.1016/j.heliyon.2023.e14454

**Published:** 2023-03-11

**Authors:** Uttam Golder, Nishat Rumaly, Mohammad Kamal Hossain, Meher Nigar

**Affiliations:** a Department of Finance and Banking, Jashore University of Science and Technology, Jashore-7408, Bangladesh; b Department of Accounting and Information Systems, Jashore University of Science and Technology, Jashore-7408, Bangladesh

**Keywords:** Asymmetric relationship, Financial progress, Remittances, Economic growth, NARDL, Bangladesh

## Abstract

Prior studies in Bangladesh examined the effects of financial progress and inward remittances on economic growth and postulated a symmetrical relationship, ignoring the potential asymmetrical relationship between variables. Therefore, this study intends to explore the asymmetrical effects of financial progress and remittances on economic growth in Bangladesh. The study used yearly time series data from 1988 to 2020 and employed the Nonlinear Autoregressive Distributed Lag (NARDL) model. Our study confirmed the asymmetrical effects of financial progress and remittances on economic growth and revealed a long-run association between the variables being studied. The study's novelties are that both positive and negative fluctuations in financial progress and remittances boost Bangladesh's economic growth. Thus, it is essential to enact policies that support financial progress by ensuring sustainable development in financial institutions and financial markets. In addition, remittances should be employed for productive purposes rather than only for consumption to improve the country's economic backbone.

## 1 Introduction

Understanding the factors that affect the economic growth of a country is indefensible for policymaking purposes. In the context of Bangladesh, it has a proven track record of rapid economic growth over the past 20 years, supported by a strong demographic dividend, robust ready-made garment (RMG) exports, resilient remittance inflows, and stable macroeconomic conditions [1]. With its steady economic growth, the country aims to meet the Sustainable Development Goals (SDGs) by 2030, develop middle-income economies by 2031, and become an emerging nation by 2041. According to Refs. [2,3], remittances and financial progress are important contributors to GDP and promote economic growth.

Financial progress, a proxy for financial development, refers to the rise of financial activity that makes the financial sector more productive by eliminating the financial recession and improving the financial structure through innovation and diversification [4]. Economic growth may be accelerated by increasing financial development and raising private sector investments that ultimately contribute to the public sector [5]. Banks, which are dominated by private entrepreneurs, have been dominating the financial sector of Bangladesh since the 1990s, and the country's economy is heavily reliant on the banking sector. It underwent a series of market-based deregulation adjustments and continued to implement cutting-edge improvements to this day [6]. However, there is still much room for growth in Bangladesh's banking sector, and the emergence of financial markets and stock exchanges is still in its infancy [7]. Secured, easy, and expanded access to financial sector credit boosts private sector investments, which in turn can significantly contribute to nations' economic growth and development [5].

Remittances deliver vital advantages to the emigrant nations and compensate for the loss of some of the workforce [8]. The flow of remittances to the receivers' countries implies repaying those nations for the brain drain specific to them through immigration [9,10]. Remittance channels more funds to recipient households, which they might utilize for increased consumption, better access to healthcare or educational opportunities, better accommodation and living arrangements, and the utilization of resources in constructive initiatives [11]. Moreover, remittances boost national savings, making it easier for new investments [12]. Bangladesh had 7.40 million migrants residing abroad in 2020 [13]. According to the World Bank [14], Bangladesh received the seventh-largest sum of money transferred by migrant workers among the top 10 receiving countries. Remittance inflows have benefited Bangladesh's international creditworthiness by alleviating the strain from the country's merchandise trade balance deficits and contributing to economic growth with significant GDP production [11].

The effects of financial progress and remittances on economic growth have been the subject of numerous studies in Bangladesh [6,7,[15], [16], [17], [18], [19], [20]]. For example [15,16,18], employed the ARDL model to examine the effects of financial progress and remittances on economic growth in Bangladesh [7]. employed the Johansen cointegration method and Granger causality test to identify the long-run relationship and the direction of the causality, and [17,19,20] employed the VECM model to understand the relationship between the variables being studied. These studies primarily focused on the long- and short-term symmetric relationships between financial development, remittances, and economic growth in Bangladesh, while ignoring any potential asymmetric relationships. Therefore, prior research on Bangladesh has not revealed whether the effects of financial progress and remittances on economic growth are asymmetric. This reveals a gap in the current body of knowledge.

The asymmetric effects of financial progress and remittances on economic growth are the subject of a growing body of research. This is because the majority of economic relationships in the real world appear to have nonlinear characteristics, and the rate of economic growth may not always be directly correlated with changes in financial progress and remittances. When financial progress and remittances are symmetrically related, their impact on economic growth is equal both when they are increasing and when they are decreasing. Studying symmetric relationships may not be perfectly adequate because the magnitude of change in GDP may differ due to the positive and negative directions of financial progress and remittance. Increasing financial progress may have a greater impact on GDP than decreasing financial progress or vice versa. Therefore, understanding the asymmetrical relationship is crucial for policymakers in Bangladesh because the government's policies will differ if the magnitude of influence is not equal on both sides of the change in financial progress and remittance. The overall economy of the country may not benefit if they are unable to demonstrate an asymmetrical relationship between financial progress, remittances, and economic growth and if the same measures of policies are implemented both during the expansion of remittances and financial progress as well as during their downturn. This fact motivates us to carry out this study. It raises a key research question: are the effects of financial progress and remittances on economic growth in Bangladesh symmetric or asymmetric?

Therefore, this research aims to examine the asymmetric effects of financial progress and remittance on the overall economic growth in Bangladesh. The study finds a positive asymmetric effect of financial progress and remittances on economic growth in Bangladesh, which is in line with expectations. Surprisingly, the study also finds that as financial progress and remittances slow, the country's economic growth speeds up. This study is the first to employ an asymmetric model, such as the Nonlinear Autoregressive Distributed Lag (NARDL) model, and to use the composite index for financial progress recommended by the IMF, which takes into account the accessibility, efficiency, and depth of both financial markets and financial institutions. The long-run consequences of a positive (negative) shock to financial progress are predicted to have a positive (positive) effect on economic growth. Additionally, if the inward remittances experience positive (negative) shocks, this will also positively (positively) impact the rate of economic growth.

The study is significant because there are a variety of factors that can have an impact on a nation's economy, including pandemics (like COVID, SARS, smallpox, etc.), domestic and global unrest, economic recession, and protracted war (e.g., the Russia-Ukraine war, the China-USA trade war, the conflict in Ethiopia, etc.). Consequently, there is a high likelihood that the systems for managing production, consumption, and the supply chain will be disrupted. Mutual funds, insurance, and pension growth could all be impacted. The banking sector may be reluctant to provide financing for production. The bank's operational costs could also increase while interest income, return on assets, and return on equity all decrease. In addition, it is possible that the depth, accessibility, and efficiency of the financial market could all decline, compounding problems in the financial sector. The depth, accessibility, and efficiency of the financial market could all decrease, which would exacerbate the problems facing the entire financial sector. In these conditions, factors like financial progress and remittances may have had an asymmetrical rather than a symmetrical impact on the country's economic growth. The policymakers of the country must comprehend the full scope of the impact. Therefore, the study is expected to contribute to the existing literature by exploring the asymmetric relationship among the variables being studied.

The rest of this study is designed as follows: Section 2 presents a detailed literature review, and Section 3 discusses methodology. The empirical results are discussed in Section 4, and Section 5 offers the conclusion and policy implications.

## 2 Literature review

This study seeks to ascertain whether financial progress and remittances have an asymmetrical impact on economic growth in Bangladesh. Economic growth is the most crucial determinant of a country's development. Bangladesh, like many other countries, is concerned about the drivers, including remittances, financial development, and trade openness, that ensure economic growth. Progress in a country's financial system is often referred to as the “blood circulation” of the economy. A country's unemployment crisis can be eased by strengthening the financial sector, which ultimately moves the nation closer to achieving its long-term goals [21]. If the financial sector is degraded, a country's economy may be muddled.

Many governments are committed to ensuring economic growth by focusing on various economic aspects. A country's economic progress can be attributed to numerous factors, both domestically and internationally. Several previous studies examined the relationship of domestic financial progress, market capitalization, remittances, openness to international trade, banking sector innovation, and foreign direct investment (FDI) sources with the ultimate economic growth of different countries [17,[22], [23], [24]]. Remittance inflow is a significant source of rising foreign cash that stabilizes the exchange rate and ensures economic growth [10]. Also, trade openness substantially determines economic growth and the number of foreign reserves. Numerous authors have emphasized the impact of several macroeconomic variables in light of their relationship to economic prosperity.

Taking data from India [25], reveals an association between financial development and economic growth by employing cointegration and causality testing models. The study also finds a symmetric long-term link between the two variables and argues that economic growth boosts financial progress, which speeds up economic growth in return [23]. analyzing data from Ghana for the period 1980–2016, examine the nonlinear impact of financial progress on economic growth and confirm the presence of a long-term asymmetric effect on growth caused by both positive and adverse effects of financial progress. The study also documents that the effect of the financial sector would determine the growth of economic development. Many other studies on Vietnam, Malaysia, and Sri Lanka also expose that financial progress has an outstanding contribution to economic growth [[26], [27]].

According to Ref. [24] economic progress and the alleviation of poverty and inequality depend heavily on remittances sourced from abroad. The study suggests policies that would implement various programs to encourage migrant workers to send money home to their families. Using data collected from Nepal for the period 1984–2014 [21], find that financial progress is positively and significantly linked to economic growth in the long- and short-term. Similarly [28], reveal in their study that financing for the private sector and securities trading implies a beneficial effect on the rise of the country's GDP. According to [29], the development of the stock market is indispensable, and ease of access to banking channels is needed to increase economic growth.

Trade openness, which indicates the global trading situation of a country relative to its GDP, is a vital determinant of economic progress [30]. documents a productive association between trade openness and economic progress. However [31], state that using trade intensity metrics to gauge openness can result in inaccurate outcomes regarding the expansion of trade. Similarly [32], posit that India's financial development has both positive and negative effects. Still, the impact of control variables like the currency rate and openness to trade is consistent with fundamental economic understanding. In Bangladesh [18], analyze data from 1987 to 2019 using a linear ARDL model to find the significance of financial improvement on the progression of the country's economy. They confirm that a dynamic financial sector ensures the economic success of the country. As with the preceding studies, many other studies [17,19,24,33] find a positive linkage between financial progress and economic growth in Bangladesh.

In contrast [34], analyze the data collected from Kenya and reveal that financial progress in the long-term period reduces economic outgrowth. The study suggests that strategies to reduce inflation, improve resource solidarity, and appropriately allocate capital are necessary to boost economic growth. Similarly [16], find a negative nexus between financial progress and economic growth in Bangladesh by examining data from 1985 to 2019. However, they reveal a long-term positive influence of infrastructure advancement and human capital on economic growth, recommending more investment in structural development and the utilization of human capital. Employing the causality testing technique and VECM method [20], examine the consequences of FDI, capital accumulation, workforce, and financial progress on the outgrowth of the economy in Bangladesh. They find that financial circumstances negatively impact GDP, indicating inadequate exposure to the banking sector.

Remittances have accounted for almost 35% of total revenue generated by exports for the past ten years and are considered the next most significant source of international earnings after the RMG sector in Bangladesh [35]. [2] examine the influence of remittance inflow on the economies of six selected countries, analyzing data from 1999 to 2013. They confirm a productive relationship between the two variables. This result is reinforced by Ref. [36] while studying the consequences of remittance inflow on the economic expansion of 36 African countries for the years 1980–2004. They find that a 10% increase in remittance inflow helps improve GDP, the proxy for economic growth, by 0.04% [10]. also examine the data of MINT countries from 1980 to 2019 to understand the influence of remittances and financial progress on the economic growth of those countries. The study uses the panel ARDL and NARDL models and reveals that financial progress and inward remittances are the two factors contributing to economic growth. However [3], reveals an adverse effect of remittances on the economic growth in Bangladesh, Pakistan, and Sri Lanka. Table 1 presents a summary of some current literature reviews.

**Table 1:** Summary of studies on the determinants of economic growth.

Authors	Country(s)/Region	Duration	Methods	Finding(s)
[29]	ASEAN Regional Forum (ARF)	1991–2011.	PCA	Positive impact of financial development on per capita economic growth in a linear direction
[31]	CEE countries	1995–2013	PSCE and LSDVC	Trade openness positively impacts economic growth in a linear way
[20]	Bangladesh	1990–2018	VECM	Financial development impacts linearly and negatively GDP in the long run
[10]	MINT nations	1980–2019	ARDL and NARDL	Financial development and remittance have both linear and nonlinear positive impacts on economic growth
[15]	Ghana	1961–2010	PCA	Positive linear impact of financial development on growth
[16]	Bangladesh	1985–2019	ARDL	Linear negative effect of financial development on economic growth
[7]	Bangladesh	1985–2014	Johansen cointegration method	Casual and linear relationship between economic growth and other indicators of financial progress
[6]	Bangladesh	1974–2012	Regression Model	Loans to the private sector have a linear and positive impact on economic growth.
[17]	Bangladesh	1977–2016	VECM	Financial development and economic growth have a long-run linear relationship
[36]	African countries	1980–2004	Random Effects and Quasi-Fixed Effects Models	Remittances have a linear and positive impact on economic growth.
[18]	Bangladesh	1987–2019	ARDL	Financial development has a positive and linear influence on economic growth
[26]	Malaysia	1960–2003	PCA	Higher economic growth follows financial development linearly
[32]	India	1996–2018	NARDL	Positive nonlinear effects of financial development on economic growth

To sum up, despite the fact that several studies in Bangladesh have attempted to link financial progress, inward remittances, and economic growth, none have used the broad-based index for financial progress. In addition, no study has yet attempted to show how financial progress and remittances asymmetrically affect economic growth. Therefore, this study aims to examine the asymmetric impact of remittances and broad-based composite financial progress on economic growth in Bangladesh.

## 3 Methodology

This section provides the methodology employed in the study.

### 3.1 Variables

This study used time-series data to examine the asymmetric effects of financial progress and inward remittances on economic growth in Bangladesh. Table 2 provides a summary of the variables employed in this study. To minimize outlier effects, GDP per capita is turned into a natural logarithm.

**Table 2:** Variables’ description summary.

Variable	Symbols	Explanation	Measurement scale
*Dependent variable:* Economic growth *Independent variables:*	GDPPC	The total output of goods and services increases over time.	GDP is a ratio of the total mid-year population of a country.
Financial progress	FP	The strength of financial institutions and financial markets of a country.	IMF broad-based index of financial development.
Inward remittances *Control variable:*	REM	Funds sent home by expatriate workforces.	Personal remittance received in current US$ as a portion of GDP.
Trade openness	TO	The total value of goods and services exported and imported.	Amount of goods and services exported and imported as a percentage of GDP.

### 3.2 Research model

**Fig. 1:**
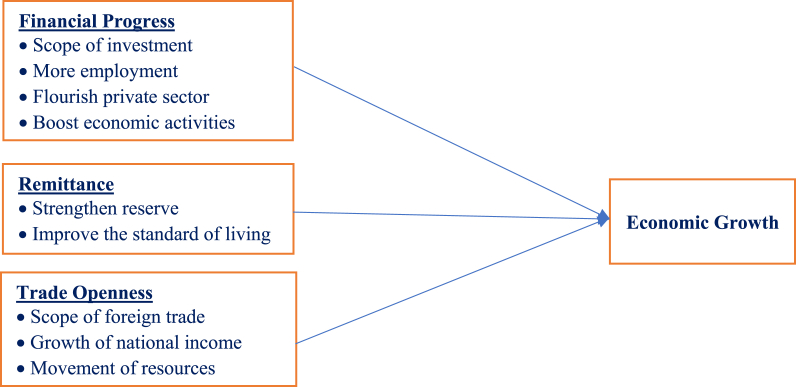
General mapping of financial progress, remittance, and trade openness impacts on economic growth.

Fig. 1 illustrates the possible influence of financial progress, inward remittances, and trade openness on the economic growth in Bangladesh. As the new broad-based index of financial development identifies the depth, access, and efficiency of financial institutions and financial markets, it ultimately encourages economic activities and affects the GDP by creating a scope of new investments, more employment, and flourishing private sectors [17]. Also, remittance is an essential macroeconomic factor that may significantly impact a country's GDP by boosting foreign reserves and improving the standard of living [11]. As for the control variable, trade openness helps increase domestic output by increasing national income, foreign trade, and mobilization of resources and thereby increasing a country's GDP [30].

### 3.3 Model specification

Following the study of [10], we use equation (1) to examine the long-term nexus of financial development and inward remittance on economic growth in Bangladesh.

(1) $${GDPPC}_{t}=\beta_{0}+\beta_{1}\left({FP}_{t}\right)+\beta_{2}\left({REM}_{t}\right)+\beta_{3}\left({TO}_{t}\right)+\varepsilon_{t}$$

Where *GDPPC, FP, REM,* and *TO* denote per capita GDP, financial progress, inward remittance, and trade openness in a given time *t*, respectively. $\beta_{i}$ characterizes the long-term parameters, and $\varepsilon_{t}$ is the disturbance term. In equation (1), variations in the independent variables are assumed to have the same effect across the whole time series, which, in all cases, may not be feasible depending on the situation. The majority of economic linkages are nonlinear. Besides, linear models and estimation methods, if employed incorrectly, may be inappropriate and lead to inaccurate policy conclusions [[37], [38], [39]]. On the other hand, the NARDL model introduced by Ref. [40] accounts for the long-term effects of the predictor variable on the predicted one and makes it possible to look at the positive and negative influences when short-term and long-term nonlinearity is taken into account through a positive and negative fractional sum breakdown of the predictor variables. These linkages between financial progress, remittances, and economic growth in Bangladesh might be studied using the functional variation of the model and can be formulated as per equation (2).

(2) $$GDPPC=\left({FP}^{+},{FP}^{-},{REM}^{+},{REM}^{-},TO\right)$$

Following the studies by Refs. [38,[41], [42], [43]] the asymmetric long-run effect of financial progress and remittance on economic growth in Bangladesh can be determined by using equation (3), which is a rewrite of equation (2).

(3) $${GDPPC}_{t}=\emptyset_{0}+\emptyset_{1}\left({FP}_{t}^{+}\right)+\emptyset_{2}\left({FP}_{t}^{-}\right)+\emptyset_{3}\left({REM}_{t}^{+}\right)+\emptyset_{4}\left({REM}_{t}^{-}\right)+\emptyset_{5}TO+\mu_{t}$$

Where $\emptyset_{i}$ indicates the coefficients of the long-run parameter to be assessed, and ${FP}_{t}^{+},{FP}_{t}^{-},{REM}_{t}^{+}\text{,}$ and ${REM}_{t}^{-}$ are positive and negative fractional sum breakdowns of *FP*, and *REM*, respectively. Using equation (1) alone is insufficient as it focuses on the long-term impact of predictors rather than the short-term impact. This study's error-correction model specification for equation (1) is given in equation (4).

(4) $$\Delta {GDPPC}_{t}=\vartheta_{0}+\sum_{j=1}^{p}\vartheta_{1j}\Delta {GDPPC}_{t-j}+\sum_{j=1}^{p}\vartheta_{2j}\Delta {FP}_{t-j}+\sum_{j=1}^{p}\vartheta_{3j}\Delta {REM}_{t-j}+\sum_{j=1}^{p}\vartheta_{4j}\Delta {TO}_{t-j}+\omega_{1}{GDPPC}_{t-1}+\omega_{2}{FP}_{t-1}+\omega_{3}{REM}_{t-1}+\omega_{4}{TO}_{t-1}+\pi_{t}$$

Where Δ signifies the difference operator in period *t*, and *p* is lag orders. The long-term fluctuation of the predictor factors on the responder variable is denoted by $\omega_{i}$, while $\sum_{j=1}^{p}\vartheta_{ij}$ estimates the short-run consequences of predictor variables on economic growth. Equation (4) predicts the symmetrical link between the anticipated variables. However, this study is focused on understanding how financial progress and remittances nonlinearly affect economic growth in Bangladesh; hence, the nonlinear cointegrating methodology is used instead. Disintegrate regression is embodied by $z_{t}=\emptyset^{+}f_{t}^{+}+\emptyset^{-}f_{t}^{-}+\pi_{t}$, where $\emptyset^{+}$ and $\emptyset^{-}$ are associated with long-run parameters and in equation (5), $f_{t}$ is a regressor vector shattered into by way of:

(5) $$f_{t}=f_{t}^{+}+f_{t}^{-}$$

Where $f^{+}$ and $f^{-}$ are the predictor variables, and are disintegrated into the sectional sum of negative and positive changes. Following Shin et al. [40], the values of ${FP}_{t}^{+},{FP}_{t}^{-},{REM}_{t}^{+}\text{,}$ and ${REM}_{t}^{-}$ can be estimated through equations (6), (7), (8), (9).

(6) $${FP}^{+}=\sum_{r=1}^{t}\Delta {FP}_{r}^{+}=\sum_{r=1}^{t}\max\left(\Delta {FP}_{r},0\right)$$

(7) $${FP}^{-}=\sum_{r=1}^{t}\Delta {FP}_{r}^{-}=\sum_{r=1}^{t}\min\left(\Delta {FP}_{r},0\right)$$

(8) $${REM}^{+}=\sum_{r=1}^{t}\Delta {REM}_{r}^{+}=\sum_{r=1}^{t}\max\left(\Delta {REM}_{r},0\right)$$

(9) $${REM}^{-}=\sum_{r=1}^{t}\Delta {REM}_{r}^{-}=\sum_{r=1}^{t}\min\left(\Delta {REM}_{r},0\right)$$

Consistent with [40], this study replaces equation (1) with equation (3) to attain the nonlinear ARDL model with unique long-run and short-run asymmetric associations that can be expressed in equation (10).

(10) $$\Delta {GDPPC}_{t}=\emptyset +\sum_{j=1}^{p}\emptyset_{j}\Delta {GDPPC}_{t-j}+\sum_{j=1}^{p}\emptyset_{j}\Delta {FP}_{t-j}^{+}+\sum_{j=1}^{p}\emptyset_{j}\Delta {FP}_{t-j}^{-}+\sum_{j=1}^{p}\emptyset_{j}\Delta {REM}_{t-j}^{+}+\sum_{j=1}^{p}\emptyset_{j}\Delta {REM}_{t-j}^{-}+\sum_{j=1}^{p}\emptyset_{j}\Delta {TO}_{t-j}+\omega_{1}{GDPPC}_{t-1}+\omega_{2}{FP}_{t-1}^{+}+\omega_{3}{FP}_{t-1}^{-}+\omega_{4}{REM}_{t-1}^{+}+\omega_{5}{REM}_{t-1}^{-}+\omega_{6}{TO}_{t-1}+\pi_{t}$$

Where, $\omega_{i}$, and $\sum_{j=1}^{p}\emptyset_{j}$ specify the coefficients of the long-run and short-run fluctuations of financial progress, remittance, and trade openness on economic growth. Moreover, the long-run effect of positive and negative fluctuations on economic growth can be estimated as ξ1=−ω2ω1, ξ2=−ω3ω1, ξ3=−ω4ω1, and ξ4=−ω5ω1.

### 3.4 Data and data summary

From 1988 to 2020, data for GDP per capita, inward remittances, and trade with no missing values were adopted from the WDI, a reliable and trustworthy source of financial information backed by the World Bank. For financial progress, this study extracts data from the IMF, where a broad-based financial development index was developed by Ref. [44]. Table 3 summarizes the study variables and shows that all the variables are normally skewed. Furthermore, the Jarque-Bera test confirms the normality of all variables tested, which are all platykurtic.

**Table 3:** Descriptive statistics.

	GDPPC	FP	REM	TO
Mean	6.6904	0.1921	5.6531	32.1740
Median	6.5968	0.1923	5.4070	30.5195
Maximum	7.4047	0.2844	10.5879	48.1109
Minimum	6.2054	0.1245	2.4649	17.6781
Std. Dev.	0.3743	0.0451	2.6456	9.0182
Skewness	0.4410	0.2109	0.4279	0.0715
Kurtosis	1.9380	1.8305	1.8080	1.9482
Jarque-Bera	2.6206	2.1253	2.9609	1.5493
Probability	0.2697	0.3455	0.2275	0.4609

### 3.5 Estimation methods

Using time-series data estimators, this work applies the NARDL model to determine the asymmetric connection between financial progress, inward remittance, and economic growth. The NARDL method offers several benefits over the traditional ARDL paradigm. For instance, it allows for the investigation of asymmetries, which contradicts the linearity assumption. Models of time series regression cover fixed parameters and consider the underlying assumption that a movement in an explanatory variable has the same influence over time. Although this may not be a faithful assumption in many circumstances, it is generalized in all cases. Conventional cointegration tests, e.g., the classic Johansen cointegration [45] and the Engle-Granger [46], imply constant adjustment over time. These may not always be feasible in practice, and the linear estimate may not always be appropriate, leading to incorrect policy formulation [37,38]. In both the long and short runs, the NARDL model investigates the potential for an asymmetric interaction between the possible effects of the predictor variable on the predicted one. Statistically, the NARDL model is comparable to the classic ARDL since it permits the inclusion of regressors with mixed orders of integration. Initial steps in NARDL model estimation include conducting the unit root test, which checks to certify that no variables are I(2). Augmented Dickey-Fuller (ADF) and Phillips Perron (PP) unit root tests are used to ensure the stationary nature of all variables in the early stage of the investigation. However, any structural break in the data series could potentially produce unreliable results. To avoid unreliable results, this study employs Zivot and Andrews' [47] unit root test to examine the possibility of a structural break occurring. As the current study finds the presence of a structural break, equation (10) turns into equation (11).

11 $$\Delta {GDPPC}_{t}\emptyset +\sum_{j=1}^{p}\emptyset_{j}\Delta {GDPPC}_{t-j}+\sum_{j=1}^{p}\emptyset_{j}\Delta {FP}_{t-j}^{+}+\sum_{j=1}^{p}\emptyset_{j}\Delta {FP}_{t-j}^{-}+\sum_{j=1}^{p}\emptyset_{j}\Delta {REM}_{t-j}^{+}+\sum_{j=1}^{p}\emptyset_{j}\Delta {REM}_{t-j}^{-}+\sum_{j=1}^{p}\emptyset_{j}\Delta {TO}_{t-j}+\sum_{j=1}^{p}\emptyset_{j}\Delta {DUM}_{t-j}++\omega_{1}{GDPPC}_{t-1}+\omega_{2}{FP}_{t-1}^{+}+\omega_{3}{FP}_{t-1}^{-}+\omega_{4}{REM}_{t-1}^{+}+\omega_{5}{REM}_{t-1}^{-}+\omega_{6}{TO}_{t-1}+\omega_{7}{DUM}_{t-1}+\pi_{t}$$

When all of the explanatory variables have been generated, the next step is to compute the partial coefficients (the increase or decrease impacts of the explanatory variables on the explained one) for each of them, namely financial progress and remittance. As a further stage in the analytical process [48], use lower and higher critical values for I(0) and I(1) to compare with the F-statistics of the bound test, and the F-statistic reveals a long-term relationship between variables and verifies cointegration if it surpasses the upper bounds. The dynamic estimation of the NARDL model and the long-term coefficients, which are also determined logically, constitute the next stage, and after that, testing the asymmetric co-integration of the variables is required. Finally, this study employs several diagnostic tests, including serial correlation, normality, and heteroskedasticity. Further, it also uses CUSUM and CUSUM-SQ tests to check the model's stability.

## 4 Results and discussions

This study necessitates that no variables be incorporated into the second order. The results conveyed in Table 4 have been cross-validated using the ADF and PP unit root tests, which reveal that, in ADF, all variables are integrated into first-order except remittances, which are integrated at the level. In PP, all the variables are integrated into the first order. Hence, the NARDL can be implemented as the investigation found no second-order unit root in any of the variables in the study.

**Table 4:** Unit root tests.

Test	GDPPC	FP	REM	TO
ADF				
I(0)	−1.2444	−3.1603	−4.2190**	−0.5836
I(1)	−4.2351**	−4.3226***	–	−4.9122***
PP				
I(0)	−1.2532	−2.4460	−1.3002	−0.5509
I(1)	−4.2175**	−4.7422***	−3.6670**	−4.8264***

Note: ***p<1%, and **p<5%. Source: Authors' computations.

Table 5 presents the outcomes of the ZA unit root test. When one potential break in the dataset is considered, the ZA test for unit roots reveals all the variables are stationary at level, and the structural shift in GDPPC, FP, REM, and TO occurred in 2002, 2004, 2006, and 2011, respectively. The ZA test revealed a structural break in GDPPC in 2002, and thus, a new dummy variable (${DUM}_{GDPPC}$) is considered in equation (11), which takes 0 until 2001 and after that, 1.

**Table 5:** Zivot-Andrews unit roots test.

Variable	t-stat	Year of break	Result
GDPPC	−4.0797*	2002	Stationary
FP	−4.5864**	2004	Stationary
REM	−4.2441**	2006	Stationary
TO	−4.6397***	2011	Stationary

Note: ***p<1%, **p<5% and * p<10%. Source: Authors' computations.

Lag is a vital aspect in determining the model's accuracy, and if too few or too much lag is set, the model might lose out on important information or overfit the model. According to this study, which relied on AIC to detect optimum lag selection, lag 2 proved to be the most beneficial. Table 6 shows the findings of bound testing results, where the F-statistic is matched with the two crucial values of the upper and lower bound. This model has an F-statistic value of 11.86, and its upper bounds at the 1% and 10% significance levels are 3.99 and 2.94, respectively. This model's F-statistic crosses the 1% and 10% critical values, confirming the variables' long-term asymmetrical association. As a result, the nonlinear ARDL model might be utilized moving ahead.

**Table 6:** Bounds test results.

Model	F-stat	Upper bound	Lower bound
GDPPC/(${FP}^{+},{FP}^{-},{REM}^{+},{REM}^{-},TO,{DUM}_{GDPPC}$)	11.86		
Critical values			
10%		2.94	1.99
5%		3.28	2.27
2.5%		3.61	2.55
1%		3.99	2.88

The optimum lag of 2 has been combined with eliminating any extraneous predictors from the model to achieve the required parameters for the study. In accordance with [42], equation (11) has been computed utilizing the general and specific processes to estimate the final model.

Table 8 reports a long-run asymmetric association between financial progress, remittances, and economic growth. Based on the findings reported in Table 7, the long-term results in Table 8 are computed. The result shows that a 1% increase in financial progress increases economic growth by 0.73%, indicating that financial progress boosts economic advancement in Bangladesh. Private sector loans put more money into the financial system, leading to more investment by private institutions and extra-economic activity in the country [18]. In addition, bank loans to the non-government sector replicate the mobilization and pooling of funds, create an atmosphere for investments, encourage foreign capital to follow, make advancements in technology, and stimulate output that adds value to a nation's GDP [10,34]. Further, the development of pension funds, mutual funds, and insurance companies, the growth of bank branches and their ATMs, the profits of banking sectors (e,g., ROA, ROE, and NIM), and the development of stock markets all help to grow the economy. However, this finding is inconsistent with that of [24], who reveal that the expansion of domestic credit to the private sector decreases the economic growth of the country.

**Table 7:** Dynamic assessment of NARDL results.

Variable	Coeff.	Std. Error	t-Stat	Prob.
C	2.9010	0.5290	5.4843	0.0001
GDPPC(−1)	−0.4690	0.0848	−5.5330	0.0001
${FP}^{+}\left(-1\right)$	0.3424	0.0935	3.6620	0.0029
${FP}^{-}\left(-1\right)$	−0.3569	0.1464	−2.4381	0.0299
${REM}^{+}\left(-1\right)$	0.0118	0.0035	3.3788	0.0049
${REM}^{-}\left(-1\right)$	−0.0293	0.0041	−7.0763	0.0000
TO	0.0022	0.0004	5.6535	0.0001
${DUM}_{GDPPC}\left(-1\right)$	0.0183	0.0078	2.3462	0.0355
dGDPPC(−1)	−0.4392	0.2201	−1.9948	0.0675
$d{FP}^{+}$	0.1386	0.0721	1.9213	0.0769
$d{FP}^{+}\left(-1\right)$	−0.1715	0.0870	−1.9700	0.0705
$d{FP}^{-}$	0.0314	0.1608	0.1953	0.8482
$d{REM}^{+}$	0.0071	0.0042	1.6634	0.1201
$d{REM}^{-}$	−0.0070	0.0031	−2.2882	0.0395
$d{REM}^{-}\left(-1\right)$	0.0134	0.0038	3.5056	0.0039
${dDUM}_{GDPPC}$	−0.0161	0.0093	−1.7356	0.1063
${dDUM}_{GDPPC}\left(-1\right)$	−0.0257	0.0076	−3.3774	0.0050

**Table 8:** Long-run asymmetric connection.

Variable	Coeff.	Std. Error	t-Stat	Prob.
${FP}^{+}$	0.7300	0.1167	6.2548	0.0000
${FP}^{-}$	−0.7610	0.2816	−2.7027	0.0181
${REM}^{+}$	0.0251	0.0040	6.2065	0.0000
${REM}^{-}$	−0.0625	0.0052	−11.9238	0.0000
TO	0.0046	0.0012	4.0073	0.0015
${DUM}_{GDPPC}$	0.0390	0.0197	1.9811	0.0691
C	6.1855	0.0213	290.6687	0.0000

The study also shows that Bangladesh's GDP rises by 0.7610% for every 1% decline in financial progress, indicating that negative shocks to financial progress promote a country's economic growth. This result is consistent with those of [10,24]. The result also reveals that the negative shock of financial progress has a greater impact on Bangladesh's economic growth than a positive one. A possible explanation for this might be that the benefits of credit distribution are significantly reduced by an excessive number of defaulted loans, lax government oversight, and the adverse selection of loan projects in the banking sector brought on by a lack of good governance. When a country's money supply contracts, it narrows the window for distributing loans, and if a bank or non-banking financial institution lacks the resources, it looks for creditworthy borrowers with fewer opportunities to have loans classified. This situation increases the likelihood that funds will be invested effectively and promotes economic progress by allowing the distribution of funds to those who are genuinely qualified to receive them. This funding, however, conflicts with the findings of [23,34], who find that poor financial development leads to increased government spending, weakens the functioning of financial institutions and slows economic growth.

As for the association between inward remittances and economic growth, the results show that a 1% increase in inward remittances accelerates the country's GDP by 0.0251% (see Table 8). The result indicates that the country's economic growth increases if the remittance grows. Our results are consistent with those of [2,3,10]. A possible explanation for this might be that remittances help to improve capital allocation and ease credit constraints in less developed countries, which helps to accelerate economic growth. Surprisingly, Table 8 shows that a 1% decrease in remittances increases GDP by 0.0625%, implying that a negative shock in inward remittances boosts economic growth in Bangladesh. The result is consistent with that of [10]. The reason for this is not clear, but it may be explained by the fact that, as many families of migrant workers have no other source of income other than remittances, most of the remittances are used to purchase food and other household essentials. These households are not concerned about the importance of saving money and investing. Also, some of them put money into constructing homes and purchasing land or apartments, which is considered unproductive. Therefore, a negative shock in remittance might lessen unproductive activities and contribute to the GDP.

We also reveal that a 1% increase in trade results in a 0.0046% increase in GDP, demonstrating that trade openness has a significant positive impact on Bangladesh's economic growth. This result is in line with that of [30]. This evidence suggests that the greater the amount of international trade (both export and import), the greater the economic growth. Finally, the dummy variable of GDPPC indicates a positive impact on the economic growth of Bangladesh.

Table 9 presents the outcomes of the Wald test to confirm the asymmetric association between financial progress, remittance, and economic growth. The long-term results shown in Table 8 indicate that there is no symmetric relationship between the variables, as evidenced by the fact that the coefficients of positive and negative fluctuations in remittance and financial progress on economic growth are not equal. The outcomes of the Wald test shown (Table 9) also confirm that financial progress and remittances have asymmetric effects on economic growth in Bangladesh.

**Table 9:** Results of the Wald test.

Variable	F-stat. [Prob]	Results of asymmetric connection
FP	16.2920 [0.0007]	Asymmetric connection exists between FP and GDP
REM	173.1694 [0.0000]	Asymmetric connection exists between REM and GDP

**Fig. 2:**
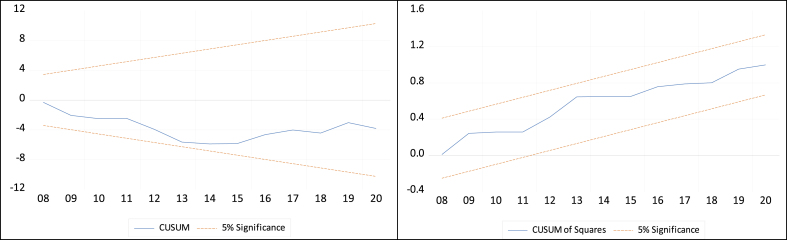
CUSUM and CUSUM-SQ tests. Source: Authors' computations.

Several diagnostic tests have been conducted to judge how well dynamic specifications work. These include the Breusch-Godfrey LM, Jarque–Bera (J–B), and Breusch-Pagan-Godfrey tests, which attempt to find the model's serial correlation, normality, and heteroscedasticity, respectively. In all cases, the p-value is greater than 5% (see Table 10), suggesting that the model has no problems with serial correlation, nonnormality, or heteroscedasticity. The Ramsey-Reset test is also used to test the model's functional form, and the p-value is greater than 5%, indicating that there is no functional error in the model. Moreover, the CUSUM and CUSUM-SQ (Fig. 2) tests measure the model's immobility and confirm its stability, as the blue lines in both cases are inside the 5% critical values of the two red lines.

**Table 10:** Diagnostic inspection.

Test	$x^{2}$ (*p-*value)/Stability	Result
Breusch-Godfrey LM	0.0515	Free from serial correlation
Jarque–Bera	0.7610	Residuals are normally distributed
Breusch-Pagan-Godfrey	0.3811	No heteroscedasticity
Ramsey RESET	0.5909	No misfunctionality
CUSUM	Stable	Ensures stability
CUSUM-SQ	Stable	Ensures stability

## 5 Conclusion and policy implication

In earlier studies in Bangladesh, linear functional specifications were used to analyze the impact of financial progress and inward remittances on overall economic growth. Depending solely on the linear symmetric association, a positive (negative) influence of financial progress and remittance on growth will create an identical effect as a negative (positive) shock to finance and remittance on economic growth. Although a simple linear relationship might illustrate the association between financial growth, remittances, and economic growth, the reality is more complex, and it could be possible that the same sorts of outcomes might not be explained by both positive and negative fluctuations in finance and remittance. In a growing nation like Bangladesh, where financial institutions and financial markets are booming, such linkages are critical and require a more in-depth investigation. Therefore, this study aims to examine the asymmetric relationship between financial progress, remittances, and economic growth in Bangladesh from 1988 to 2020. The study employed the NARDL model to find an asymmetric link between the variables being studied.

Consistent with expectations, the study finds an asymmetric effect of financial progress and remittances on economic growth in Bangladesh. More specifically, economic growth expands when there is a positive shock in financial progress and remittances. Surprisingly, this study finds that as financial progress and remittances slow, the country's economic growth accelerates. This study also explores a positive link between trade and economic growth, suggesting that more openness to international trade creates new avenues for economic growth in Bangladesh.

We made some policy implications to strengthen economic growth in Bangladesh. Banking-based financial progress is essential for economic growth; however, improper management of loans may reduce the usefulness of credit expansion, and efficient resource allocation, which may halt economic growth. Therefore, effective financial laws must be enacted, and good governance must also be ensured in the banking sector. Remittances are currently being used to invest in non-productive areas. Therefore, remittances should be directed toward sectors that support production and create jobs instead.

This study, however, is not free from limitations. For example, this study has not considered the impact of other macroeconomic factors, such as tax, inflation, and employment levels. Additionally, this study is limited to Bangladesh. Further research could be conducted by taking into account other macroeconomic factors and broadening the scope by conducting a cross-country study.

## Author contribution statement

Mohammad Kamal Hossain: Conceived and designed the experiments; Performed the experiments; Wrote the paper. Uttam Golder: Analyzed and interpreted the data; Contributed reagents, materials, analysis tools or data. Nishat Rumaly: Performed the experiments. Meher Nigar: Conceived and designed the experiments.

## Funding statement

This research did not receive any specific grant from funding agencies in the public, commercial, or not-for-profit sectors.

## Data availability statement

Data will be made available on request.

## Declaration of competing interest

The authors declare no conflict of interest.
